# Urine proteome of autosomal dominant polycystic kidney disease patients

**DOI:** 10.1186/1559-0275-9-13

**Published:** 2012-12-11

**Authors:** Magda Bakun, Mariusz Niemczyk, Dominik Domanski, Radek Jazwiec, Anna Perzanowska, Stanislaw Niemczyk, Michal Kistowski, Agnieszka Fabijanska, Agnieszka Borowiec, Leszek Paczek, Michal Dadlez

**Affiliations:** 1 Mass Spectrometry Laboratory, Institute of Biochemistry and Biophysics, Polish Academy of Sciences, Pawinskiego 5A, 02-106, Warsaw, Poland; 2 Department of Immunology, Transplant Medicine and Internal Medicine, Medical University of Warsaw, Warsaw, Poland; 3 Department of Internal Diseases, Nephrology and Dialysis; Military Institute of Medicine, Warsaw, Poland; 4 Department of Biology, Warsaw University, Warsaw, Poland

**Keywords:** ADPKD, Urine proteome, Differential proteomics, Mass spectrometry, iTRAQ, MRM

## Abstract

**Background:**

Autosomal dominant polycystic kidney disease (ADPKD) is responsible for 10% of cases of the end stage renal disease. Early diagnosis, especially of potential fast progressors would be of benefit for efficient planning of therapy. Urine excreted proteome has become a promising field of the search for marker patterns of renal diseases including ADPKD. Up to now however, only the low molecular weight fraction of ADPKD proteomic fingerprint was studied. The aim of our study was to characterize the higher molecular weight fraction of urinary proteome of ADPKD population in comparison to healthy controls as a part of a general effort aiming at exhaustive characterization of human urine proteome in health and disease, preceding establishment of clinically useful disease marker panel.

**Results:**

We have analyzed the protein composition of urine retentate (>10 kDa cutoff) from 30 ADPKD patients and an appropriate healthy control group by means of a gel-free relative quantitation of a set of more than 1400 proteins. We have identified an ADPKD-characteristic footprint of 155 proteins significantly up- or downrepresented in the urine of ADPKD patients. We have found changes in proteins of complement system, apolipoproteins, serpins, several growth factors in addition to known collagens and extracellular matrix components. For a subset of these proteins we have confirmed the results using an alternative analytical technique.

**Conclusions:**

Obtained results provide basis for further characterization of pathomechanism underlying the observed differences and establishing the proteomic prognostic marker panel.

## Background

Autosomal dominant polycystic kidney disease (ADPKD) is an inherited disorder affecting 1 in 1000 people and responsible for 10% of cases of the end stage renal disease (ESRD). Apart from renal manifestations, changes in other organs may be present, including a.o. liver cysts and intracranial aneurysms. The disease is divided into 2 types based on mutated gene (PKD1 in type 1 - 85% of cases, and PKD2 in type 2). The type of the mutation has prognostic significance, as the average age of ESRD depends on the type of the disease and amounts to 53 years in type 1, and 69 years in type 2 [1].

As potential therapeutic methods for ADPKD are extensively tested in clinical trials [2-5], there is need for tools which enable early diagnosis and monitoring of therapy, especially non-invasive tests which would substitute kidney biopsy. Evaluation of changes in the peptidome and/or proteome may provide required information of pathophysiologic and clinical significance and may allow to establish future diagnostic or prognostic tools [6]. Urine, as well-accessible compartment, seems to be an ideal material for the search of a non-invasive prognostic and therapy monitoring tests in case of renal diseases. However, before urine proteome or peptidome markers become clinically useful, the urine proteome itself must be thoroughly characterized in a process of intense multi-stage research comparing different sample processing and analysis experimental laboratory settings. The aim of our research was to apply an in-depth proteomic bottom-up methodology to characterize the urinary proteome of ADPKD population in comparison to healthy controls.

Literature data concerning descriptive proteomics in ADPKD patients are limited. Mason *et al.* reported the proteomic analysis of four samples of cyst fluid obtained postoperatively from excised kidneys in patients with ESRD due to ADPKD [7]. Kistler *et al.*[8] were the first who attempted to identify the urinary biomarker profile of ADPKD, focusing on the low molecular (<15 kDa) proteome fraction. It was thus of interest to explore other sections of the proteome in search of the differences between ADPKD and control samples. In the present study we have analyzed the proteome of the retentate of the urine filtration on 10 kDa filters. Urine samples were collected from 30 patients and carefully matched 30 healthy controls selected without introducing any bias as to the age and sex of the subjects in the aim to obtain possibly general conclusions at the present stage of the research. To obtain best possible coverage of the urine proteome for relative quantitation the MS analysis was preceded by a two dimensional tryptic peptides separation, the first dimension being isoelectrofocusing (IEF) and the second dimension – reversed phase liquid chromatography (LC). In result a list of more than 1400 proteins was established, represented by more than two peptides and subjected to comparative analysis yielding a set of 155 proteins the levels of which were different in ADPKD and control samples.

## Results

The proteome of urine collected from 30 ADPKD patients and 30 healthy subjects was compared using a combined IEF-LC-MS-MS/MS relative quantitation of iTRAQ labeled tryptic peptides. From each sample an equal amount of total protein obtained in urine retentate, after filtration on >10 kDa cutoff filters, was used for the analysis. This allowed to normalise the sample set with respect to different levels of dilution of proteome in each sample and to compare the proteome composition. After tryptic digestion peptides were subjected to iTRAQ labeling and IEF separation yielding 26 fractions, each analysed in a separate LC-MS-MS/MS run. IEF separation substantially increases the final protein coverage. However, the separate analysis of 60 samples including IEF step would require more than 1500 LC-MS-MS/MS runs which is not practical. To overcome this difficulty and retain an in depth insight into urine proteome we have used a partial pooling strategy. A set of 30 ADPKD samples was divided into 3 subsets, containing 10 samples each, which were pooled into three Disease Pooled Samples (DPS I, II and III). Similarly, control set was divided into three Control Pooled Samples (CPS I, II and III) retaining age and sex matching within the subsets. In addition two technical replicates of each DPS or CPS was prepared, further denoted A or B to assess the intragroup technical variability.

**Figure 1:**
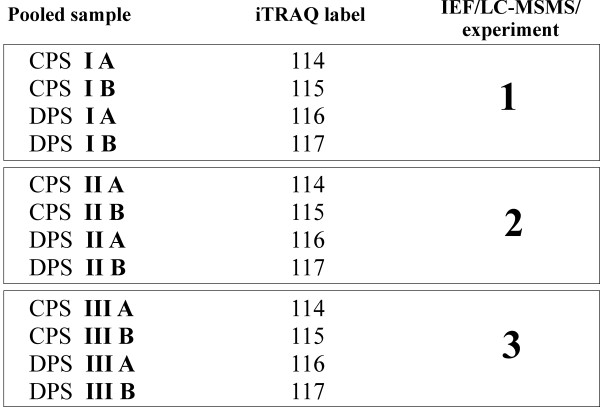
**The study design.** Combining 12 pooled samples into 3 IEF strips analysed in three LC-MS/MS experiments.

**Figure 2:**
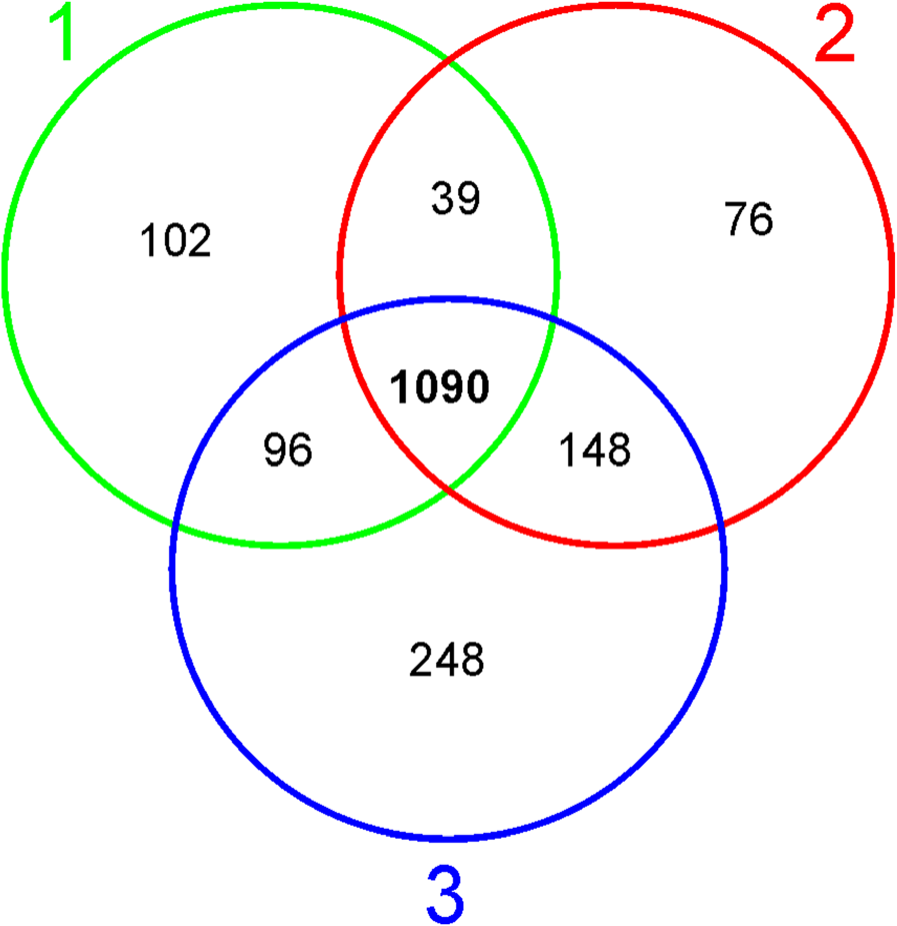
**Results of qualitative analysis – a Venn diagram representing the number of proteins identified by two or more peptides in three biological replicates of iTRAQ experiment – three IEF gel strips.** 1090 proteins are common in all three experiments.

In result 4-plex iTRAQ labeled peptides from three replicates of pooled control and ADPKD samples, each of them represented by two technical replicates, were analyzed during IEF-LC-MS-MS/MS analysis of three IEF strips, as described in Methods section and Figure 1. For each of IEF strips the two pairs of control pooled samples, for instance CPS IA, and CPS IIA, and two disease pooled samples, for instance DPS IA and DPS IIA were mixed and subjected to IEF separation. IEF strips were cut into ca. 26 sections. Labeled peptides were eluted from each of the IEF strip sections and subjected to separate LC-MS-MS/MS runs. In result of qualitative analysis (peptide and protein identification) in each of the three IEF-LC-MS-MS/MS experiments 1327/1353/1582 proteins, respectively, were identified, each represented by more than two peptides, as shown in Table 1 and Figure 2. One-peptide hits were not taken into account in further quantitative analysis.

**Table 1:** Number of identified peptides and proteins in three replicates of iTRAQ experiment on pooled samples

**Replicate (IEF gel strip)**	**Number of peptides (accepted PSM’s)**	**Number of proteins (proteins ≥ 2 peptides)**
1	9530	2430 (1327)
2	9814	2637 (1353)
3	11329	2810 (1582)

Qualitative results (protein lists) from three IEF-LC-MS-MS/MS experiments were combined, resulting in a dataset with all 1700 proteins identified by at least two peptides. Within this dataset protein identifications based on identical peptide sets were again grouped and each group was treated as a single protein cluster in further processing. Quantitative analysis was performed, as described in Methods section, with proteins represented by two or more peptides for which it was possible to calculate a protein ratio in at least one of IEF-LC-MS-MS/MS experiments. The final combined protein list accepted for quantitation contained 1413 proteins. 1090 out of these proteins are common for all replicates of the experiment.

**Figure 3:**
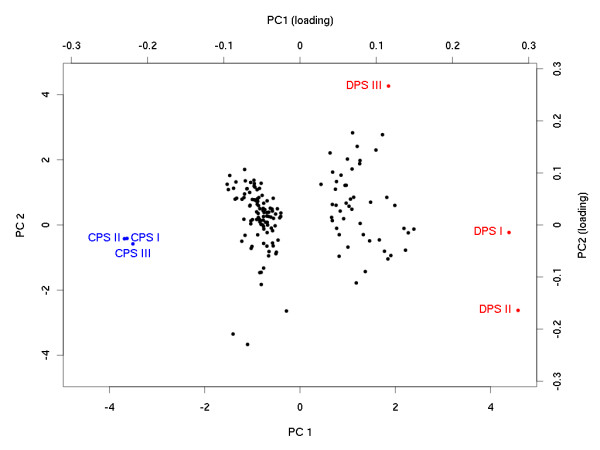
**Principal Component Analysis of the three pooled biological replicates of control (CPS I, II, and III) and disease (DPS I, II and III) samples based on 155 proteins (grey dots) indicated in the analysis as differentially populated between control and ADPKD samples.** The analysis shows a good separation of control samples from ADPKD samples along the first component axis. Note a high similarity of control samples. Normalized heights of iTRAQ peptide signals (averaged over the corresponding protein cluster) were used as features in the PCA analysis. Values of each biological replicate is an average over the two technical replicates.

The statistical analysis of the quantitative results of the three IEF-LC-MS-MS/MS experiments revealed 155 proteins that were differently populated (with q < 0.05) in the urine of ADPKD patients as compared to healthy controls. 148 of them were identified in each of the IEF-LC-MS-MS/MS experiment, 7 – in two replicates. The Differential Protein List (DPL) is presented in Table 2. The differences in protein levels (protein ratio) can be substantial, exceeding 5-fold in some cases. Among DP’s, 103 proteins were downregulated, and 52 were upregulated in ADPKD. Principal Component Analysis of the results of this experiment (Figure 3) shows a very good separation of the two study groups along the first component axis.

**Table 2:** Differential Protein List. Proteins of different level in the urine of ADPKD patients compared to healthy controls. Ratio is given as ADPKD/Control

**no**	**protein**	**qvalue**	**ratio**	**peptides**	**description**
1	P01019	0.00003	1.7	33	Angiotensinogen
2	P01008	0.00003	2.01	28	Antithrombin-III
3	P01023	0.00003	3.16	62	Alpha-2-macroglobulin
4	Q9NZP8	0.00003	0.59	23	Complement C1r subcomponent-like protein
5	Q93088	0.00003	0.55	23	Betaine--homocysteine S-methyltransferase 1
6	Q16769	0.00003	0.51	24	Glutaminyl-peptide cyclotransferase
7	P02753	0.00003	2.65	11	Retinol-binding protein 4
8	P19013	0.00003	0.3	16	Keratin, type II cytoskeletal 4
9	P19835	0.00003	0.54	27	Bile salt-activated lipase
10	P00746	0.00003	5.44	14	Complement factor D
11	P12109	0.00003	0.53	43	Collagen alpha-1(VI) chain
12	P33908	0.00003	0.48	25	Mannosyl-oligosaccharide 1,2-alpha-mannosidase IA
13	Q8WZ75	0.00003	0.41	16	Roundabout homolog 4
14	P02774	0.00003	1.78	24	Vitamin D-binding protein
15	P43652	0.00003	1.7	20	Afamin
16	Q8N307	0.00003	0.44	9	Mucin-20
17	Q9HCU0	0.00003	0.52	15	Endosialin
18	P02787	0.00003	1.84	70	Serotransferrin
19	P07602	0.00003	2.74	23	Proactivator polypeptide
20	P02679	0.00003	2.89	9	Fibrinogen gamma chain
21	P02763	0.00003	1.94	45	Alpha-1-acid glycoprotein 1
22	P00915	0.00003	3.21	12	Carbonic anhydrase 1
23	P02675	0.00003	2.17	14	Fibrinogen beta chain
24	Q13867	0.00003	0.38	10	Bleomycin hydrolase
25	P19823	0.00003	1.77	26	Inter-alpha-trypsin inhibitor heavy chain H2
26	P02647	0.00003	3.89	26	Apolipoprotein A-I
27	Q5SZK8	0.00003	0.6	22	FRAS1-related extracellular matrix protein 2
28	Q16270	0.00003	0.45	13	Insulin-like growth factor-binding protein 7
29	P36955	0.00003	2.51	25	Pigment epithelium-derived factor
30	P06727	0.00003	4.19	39	Apolipoprotein A-IV
31	P01024	0.00003	2.45	94	Complement C3
32	P61769	0.00003	3.69	21	Beta-2-microglobulin
33	Q6EMK4	0.00003	0.46	26	Vasorin
34	P20930	0.00003	0.51	33	Filaggrin
35	P02768	0.00003	1.69	191	Serum albumin
36	P01009	0.00003	1.64	99	Alpha-1-antitrypsin
37	P04114	0.00003	3.34	14	Apolipoprotein B-100
38	P31944	0.00003	0.44	15	Caspase-14
39	P04264	0.00003	1.81	44	Keratin, type II cytoskeletal 1
40	P24592	0.00003	5.08	3	Insulin-like growth factor-binding protein 6
41	O00533	0.00003	0.51	17	Neural cell adhesion molecule L1-like protein
42	P01133	0.00003	0.59	78	Pro-epidermal growth factor
43	P04180	0.00003	0.56	16	Phosphatidylcholine-sterol acyltransferase
44	P0C0L5	0.00003	1.4	83	Complement C4-B
45	P02652	0.00003	1.92	12	Apolipoprotein A-II
46	P05154	0.00003	0.38	38	Plasma serine protease inhibitor
47	P00738	0.00003	2.21	26	Haptoglobin
48	P08582	0.00003	0.54	20	Melanotransferrin
49	P05546	0.00003	2.32	13	Heparin cofactor 2
50	Q9UBC9	0.00006	0.49	18	Small proline-rich protein 3
51	P15151	0.00008	0.55	19	Poliovirus receptor
52	P04196	0.00008	1.81	14	Histidine-rich glycoprotein
53	P02766	0.00008	1.88	11	Transthyretin
54	P05090	0.00013	0.46	12	Apolipoprotein D
55	Q8NBJ4	0.00023	0.52	14	Golgi membrane protein 1
56	P04746	0.00025	0.7	84	Pancreatic alpha-amylase
57	P39059	0.00029	0.64	17	Collagen alpha-1(XV) chain
58	Q9UN70	0.00038	0.6	17	Protocadherin gamma-C3
59	P08473	0.00049	0.59	27	Neprilysin
60	Q00887	0.00069	5.04	4	Pregnancy-specific beta-1-glycoprotein 9
61	P78380	0.00082	0.56	10	Oxidized low-density lipoprotein receptor 1
62	Q99574	0.00082	0.59	20	Neuroserpin
63	P02790	0.00083	1.56	41	Hemopexin
64	Q99972	0.00134	2.39	8	Myocilin
65	P19827	0.00134	1.39	13	Inter-alpha-trypsin inhibitor heavy chain H1
66	Q6V0I7	0.00142	0.51	14	Protocadherin Fat 4
67	P02144	0.00175	3.59	5	Myoglobin
68	P55287	0.0019	0.63	21	Cadherin-11
69	Q9UQ72	0.00203	5.92	3	Pregnancy-specific beta-1-glycoprotein 11
70	P55290	0.00212	0.67	25	Cadherin-13
71	Q8IYS5	0.00221	0.55	12	Osteoclast-associated immunoglobulin-like receptor
72	Q9H8L6	0.00255	0.58	12	Multimerin-2
73	Q9NY97	0.00271	0.44	6	UDP-GlcNAc:betaGal beta-1,3-N-acetylglucosaminyltransferase 2
74	O43505	0.0032	0.52	9	N-acetyllactosaminide beta-1,3-N-acetylglucosaminyltransferase
75	P08571	0.00376	0.64	23	Monocyte differentiation antigen CD14
76	Q15828	0.00399	1.85	10	Cystatin-M
77	Q8TF66	0.00411	0.46	6	Leucine-rich repeat-containing protein 15
78	P02538	0.00417	0.57	38	Keratin, type II cytoskeletal 6A
79	P40197	0.00467	0.46	6	Platelet glycoprotein V
80	P00751	0.00492	1.66	16	Complement factor B
81	Q9BRK3	0.0055	0.67	19	Matrix-remodeling-associated protein 8
82	Q14393	0.00553	0.56	10	Growth arrest-specific protein 6
83	Q13508	0.00569	2.49	11	Ecto-ADP-ribosyltransferase 3
84	Q8IUL8	0.00569	0.61	14	Cartilage intermediate layer protein 2
85	P22105	0.00569	0.75	58	Tenascin-X
86	P54710	0.00569	0.4	4	Sodium/potassium-transporting ATPase subunit gamma
87	P07737	0.00581	2.42	5	Profilin-1
88	Q12860	0.00584	0.69	27	Contactin-1
89	Q86UN3	0.00622	0.61	8	Reticulon-4 receptor-like 2
90	P50995	0.00632	0.59	11	Annexin A11
91	P11597	0.00632	0.51	7	Cholesteryl ester transfer protein
92	Q9BRK5	0.00684	0.58	8	45 kDa calcium-binding protein
93	P35555	0.00684	0.72	15	Fibrillin-1
94	P55285	0.007	0.59	11	Cadherin-6
95	O75223	0.0075	0.63	8	Gamma-glutamylcyclotransferase
96	O75368	0.00794	2.03	5	SH3 domain-binding glutamic acid-rich-like protein
97	P51654	0.008	0.57	12	Glypican-3
98	Q9UKU9	0.00801	0.61	10	Angiopoietin-related protein 2
99	O43155	0.00801	0.59	12	Leucine-rich repeat transmembrane protein FLRT2
100	P05451	0.00803	1.7	13	Lithostathine-1-alpha
101	P29622	0.00882	0.57	14	Kallistatin
102	P02751	0.01028	0.8	90	Fibronectin
103	P98160	0.01045	0.75	115	Basement membrane-specific heparan sulfate proteoglycan core protein
104	P01031	0.01113	1.9	8	Complement C5
105	P20774	0.01175	2.5	4	Mimecan
106	Q5IJ48	0.01317	0.51	7	Crumbs homolog 2
107	Q16661	0.01319	3.75	3	Guanylate cyclase activator 2B
108	P49221	0.01365	0.67	12	Protein-glutamine gamma-glutamyltransferase 4
109	P22792	0.01403	0.64	13	Carboxypeptidase N subunit 2
110	P16444	0.0143	0.66	25	Dipeptidase 1
111	P35052	0.01447	0.68	16	Glypican-1
112	O14498	0.0148	0.63	12	Immunoglobulin superfamily containing leucine-rich repeat protein
113	O94910	0.01483	0.6	8	Latrophilin-1
114	P02511	0.01487	0.49	6	Alpha-crystallin B chain
115	Q9NRX4	0.01598	0.68	10	14 kDa phosphohistidine phosphatase
116	Q9BY67	0.01829	0.68	26	Cell adhesion molecule 1
117	P19022	0.01885	0.65	21	Cadherin-2
118	P27169	0.01891	3.48	2	Serum paraoxonase/arylesterase 1
119	Q7Z5N4	0.01891	0.61	7	Protein sidekick-1
120	O00391	0.01951	0.72	33	Sulfhydryl oxidase 1
121	Q8TB96	0.01951	0.56	6	T-cell immunomodulatory protein
122	P08603	0.01953	1.65	11	Complement factor H
123	O75340	0.02011	0.56	6	Programmed cell death protein 6
124	Q8IZF2	0.02011	0.57	9	Probable G-protein coupled receptor 116
125	Q08174	0.02013	0.65	30	Protocadherin-1
126	Q9UGT4	0.02173	0.66	23	Sushi domain-containing protein 2
127	Q15223	0.02277	0.6	7	Poliovirus receptor-related protein 1
128	P09467	0.02277	0.75	17	Fructose-1,6-bisphosphatase 1
129	P10253	0.02277	0.75	108	Lysosomal alpha-glucosidase
130	P04745	0.02277	0.47	5	Alpha-amylase 1
131	P15144	0.0253	0.71	73	Aminopeptidase N
132	Q8N271	0.02628	0.65	14	Prominin-2
133	Q9HBB8	0.02658	0.6	11	Mucin and cadherin-like protein
134	P21266	0.02672	0.65	5	Glutathione S-transferase Mu 3
135	P30530	0.0298	0.62	16	Tyrosine-protein kinase receptor UFO
136	Q08554	0.03104	3.06	2	Desmocollin-1
137	P20472	0.03378	0.52	5	Parvalbumin alpha
138	P13598	0.03484	0.63	7	Intercellular adhesion molecule 2
139	P10643	0.03556	2.02	5	Complement component C7
140	P43121	0.03723	0.65	14	Cell surface glycoprotein MUC18
141	Q08380	0.03793	0.73	41	Galectin-3-binding protein
142	O75487	0.0383	0.72	9	Glypican-4
143	O75339	0.04059	0.49	5	Cartilage intermediate layer protein 1
144	P02649	0.04059	0.69	18	Apolipoprotein E
145	P34059	0.04062	0.73	17	N-acetylgalactosamine-6-sulfatase
146	P35318	0.04219	0.49	4	ADM
147	Q12794	0.04254	0.58	7	Hyaluronidase-1
148	A9Z1Y9	0.04254	2.01	3	Thymosin beta-4-like protein 6
149	O14773	0.04437	0.69	22	Tripeptidyl-peptidase 1
150	O75015	0.04588	0.6	9	Low affinity immunoglobulin gamma Fc region receptor III-B
151	P30711	0.04618	0.4	2	Glutathione S-transferase theta-1
152	Q14894	0.04647	0.62	6	Mu-crystallin homolog
153	O43895	0.04733	0.68	18	Xaa-Pro aminopeptidase 2
154	Q9NQS3	0.04733	0.59	10	Poliovirus receptor-related protein 3
155	P04792	0.04848	0.59	7	Heat shock protein beta-1

**Figure 4:**
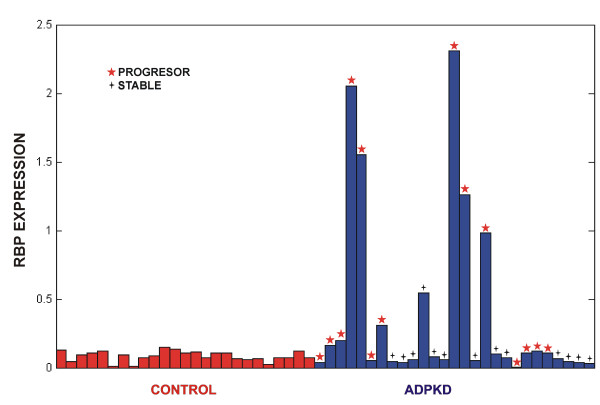
**Retinol-binding protein (RBP) levels as measured by MRM technique in a set of 27 ADPKD/25 healthy control samples.** Note large differences in protein levels within ADPKD group and correlation of its high levels with progressor status of the patient (denoted by asterisks).

DPL was obtained as a result of pooling experiment and this approach allowed for in-depth (>1000 proteins) quantitative analysis of urine proteome. However, upon pooling the levels of proteins are averaged and the information on the variability of the amount of the protein among individual samples is lost. Therefore, to test the pooling experiment results using an alternative analytical approach (Multiple Reaction Monitoring - MRM), we have carried out the analysis of individual ADPKD and control samples for a subset of proteins from DPL. For this purpose a new set of samples (27 ADPKD *vs.* 25 healthy controls) was collected. Initially, a subset of 17 proteins from DPL, represented by the largest number of peptides was selected for MRM analysis. The number of proteins for MRM experiment is limited by the number of peptides that can be analysed in parallel in a single experiment. For these proteins their natural abundance peptides were searched for in urine control samples. Satisfactory results were obtained for 9 (represented by 14 peptides) out of 17 proteins, due to insufficient sensitivity for 8 remaining proteins. Next, 14 stable isotopically labeled (SIS internal standards) peptides were synthesized. Using SIS peptides the MS parameters for MRM experiment were optimised for each peptide. Comparison of the results of the MRM quantitation with the results of iTRAQ pooling experiment for these 9 proteins is shown in Table 3. For 8 proteins their upregulation in ADPKD was in agreement with the results of the pooling experiment, however for one protein (Cystatin-M) the *q*-value (0.13) exceeded the threshold of 0.05 making this result insignificant. For still another protein (Proactivator polypeptide) MRM results for the representing peptide EIVDSYLPVILDIIK indicate its smaller level in ADPKD whereas in pooling experiment the level averaged over 19 peptides was larger in ADPKD. This result is difficult to explain since the same peptide EIVDSYLPVILDIIK in iTRAQ pooling experiment shows increased level in ADPKD, so for this protein MRM does not confirm results from pooling analysis. However, for 8 out of 9 proteins the results of both approaches are in full qualitative agreement. On the quantitative level the agreement between the two methods in the case of majority of proteins is good, only for 2 proteins the ratio differences are larger (for Retinol binding protein (RBP) ratio 4.6 for MRM and 2.65 for iTRAQ). It has to be taken into account that the ratios are calculated in both methods using a different set of peptides, usually much larger for iTRAQ. These peptides may represent different regions of protein sequence and some of them may originate from proteolytic protein fragments, quite probable in urine proteome and not from intact proteins, which may justify the observed differences on quantitative level. An alternative explanation in case of RBP comes from higher variability level of this protein within ADPKD group, as illustrated in Figure 4. It shows that the upregulation of an average RBP level in ADPKD originates from a subset (6 samples out of 27) of ADPKD samples in which the level of the protein is much larger (even by a factor of 25) than in remaining ADPKD samples, for which the levels are similar to control. Thus the average value in pooling experiment might easily be shifted by a single sample of exceptionally large content of RBP. Interestingly, the RBP levels correlate strongly with the progressor status of the patient, as illustrated by asterisks in Figure 4. This effect however requires further studies.

**Table 3:** **Comparison of the results of the MRM quantitation with the results of iTRAQ pooling experiment for 9 proteins. Protein ratios along with *q*-values are given**

	**PROTEIN**	**MRM**		**iTRAQ**		
		**ratio**	**p-value**	**ratio**	**q-value**	**peptides**
1.	Antithrombin-III	3.56	0.0001	2.01	0.00003	28
2.	Apolipoprotein A-IV	3.65	0.001	4.19	0.00003	39
*3.*	Complement C3	2.54	0.001	2.45	0.00003	94
4.	Histidine-rich glycoprotein	1.79	0.002	1.81	0.00008	14
5.	Proactivator polypeptide	0.55	0.002	2.74	0.00003	23
6.	Myocilin	1.99	0.007	2.39	0.00134	8
7.	Retinol-binding protein 4	4.6	0.015	2.65	0.00003	11
8.	Transthyretin	2.87	0.025	1.88	0.00008	11
9.	Cystatin-M	1.64	0.129	1.85	0.00399	10

## Discussion

Urine proteome is thought to contain renal disease fingerprints, but the pathology-related urine proteomics is still in its infancy. For ADPKD one study [8] was published in which a low molecular weight proteome fraction was studied and a set of potential disease markers was proposed. However, the most successful approach of global proteomic analyses of the total proteome, combining multiple steps of separation preceding quantitative mass spectrometry was not yet carried out for ADPKD urine samples. To fill this gap, in our approach we have combined iTRAQ based quantitation with peptide isoelectrofocusing and reversed phase separation coupled with MS to obtain an in-depth urine proteome coverage of quantitative analysis of ADPKD *vs.* control sample set.

Qualitative analysis – combined from three IEF-LC-MS-MS/MS experiments peptide identification brought a list of 14429 peptides assigned to proteins, corresponding to 1700 proteins, each identified by at least two peptides (Additional file 1). The median number of peptides per protein was 9.34. This list compares well with other attempts of qualitative characterization of human urine proteome in which the overall number of proteins depends strongly on the number of peptide/protein pre-fractionation steps used. 808 proteins were detected when the only separation step was LC preceding MS [9]. Adding 1D SDS PAGE separation step increased this number to 1102 [10] or 1543 [11] proteins represented by at least two peptides. Application of multidimensional separation strategy was shown to yield 2362 proteins [12], but the other group reports only 991 proteins [13]. Pairwise comparison of common proteins detected in our work yields 972 common proteins with Adachi [11], and 975 with 1823 proteins (including one-peptide hits) found by Li [13]. The number of common proteins detected in three publications [10,11,13] was compared in Figure 2 in Marimuthu's paper [10] yielding 658 common proteins of which 582 were detected in our work. This number correlates well with 587 proteins named “core urinary proteins” commonly detected in a large set of urine samples [9]. In conclusion our dataset represents very well core urinary proteins, however the number of unique proteins found in this work is also high, indicating that the urine proteome complexity is far from being explored in-depth.

In a quantitative analysis a list of proteins (DPL) differentiating ADPKD *vs.* healthy control samples has been established. The partial pooling experiment indicated a list of 155 proteins of different level in the urine of ADPKD patients compared to healthy subjects. We have found alterations in the complement system, apolipoproteins, group of serine protease inhibitors, several growth factors, collagen chains, extracellular matrix components, transmembrane proteins, and many others. Many of them have never been linked to ADPKD in previous studies. Additionally, our results confirm the alterations observed in animal models, concerning, for example, apolipoproteins [14]. Some proteins included in DPL have previously been linked to the progression of cystic kidney disease, for example CD14 molecule [15].

In our study the application of a pre-separation of peptides by IEF and the analysis of 26 fractions of each gel allowed to greatly increase the number of proteins that could be subjected to quantitation. However, each IEF-LC-MS-MS/MS experiment required 26 LC-MS-MS/MS runs corresponding to 78 hours of spectrometer time, so it could not be carried out separately for 60 samples due to exceedingly long time of the analysis required (4500 hours, nearly 200 days of spectrometer time would be required). This justified the pooling approach which combined the information contained in all samples and allowed its in-depth analysis in a reasonable time. However, when the protein ratios are compared after pooling the information on the scatter of protein ratios among the individual, pooled samples is lost, and the statistical validity of obtained differences cannot be properly assessed. For that reason we have used MRM technique for a subset of nine DPL proteins, which confirmed the results of the pooling experiment, only for one protein the confirmatory analysis was not successful. In general the differential list obtained from pooling experiment is thus a candidate list, each protein of interest from the list has to be measured in individual samples in a separate experiment by an independent method.

Only a few cases of proteomic analysis of ADPKD tissue samples can be found in the literature. Mason *et al.* reported the proteomic analysis of four samples of cyst fluid obtained postoperatively from excised kidneys in patients with ESRD due to ADPKD [7]. The authors identified 44 proteins that were found in at least two cysts and might be of mechanistic or diagnostic interest in ADPKD. Similarly to our results, the list of these proteins included complement factors, apolipoprotein A-I, pigment epithelium-derived factor (PEDF) and others. However, the potential diagnostic utility of cyst fluid proteomics is highly limited, and in our opinion, it is the urine that may become the diagnostic material in clinical practice.

Kistler *et al.* were the first who attempted to identify the urinary biomarker profile of ADPKD [8]. Due to application of CE-MS technology the range of molecular masses under study was thus limited to less than 15 kDa, whereas in our work proteins of masses larger than 10 kDa were studied. This explains the differences in the lists of differentiating proteins which in case of Kistler *et al.* were limited mainly to collagen fragments and uromodulin peptides. Therefore, our DPL may be regarded as a complete list of ADPKD-specific urinary proteins, independent on kidney function.

Our results provide the first step of the analysis, specific DPL proteins of interest should be now verified by a targeted analysis on non-pooled samples on much wider sample sets. Moreover, the specificity of these results should be determined in studies including patients with chronic kidney disease of distinct origin. Additionally, it should be determined whether the type of mutation (PKD1 or PKD2) impacts the proteome. Finally, methods of sample collection and preparation, laboratory procedures, and data analysis must be optimized. After verification, our results may in future serve as a basis for mechanistic studies and, therefore, may ultimately lead to discovery of new therapeutic targets in ADPKD. Additionally, the set of urinary biomarkers may be used in the future for early diagnosis of ADPKD.

## Conclusions

The urine proteome of ADPKD patients differs significantly from the urine proteome of healthy subjects and may become the clinical tool used for early diagnosis of ADPKD. The pathophysiological informations obtained in presented study may become a basis for the development of new therapies.

## Methods

### Urine samples

Thirty ADPKD patients diagnosed with abdominal ultrasound [16] were enrolled into the study group. The control group consisted of 30 healthy volunteers matched according to the sex and age. The demographic data of both groups are summarized in Table 4. The inclusion criteria for the study group were the diagnosis of ADPKD and age ≥18 years. The inclusion criteria for the control group included: absence of ADPKD, age ≥18 years, and body mass index (BMI) between 21 and 26. The exclusion criteria for both groups included especially: current infection of urinary tract, macroscopic hematuria, diabetes mellitus, malignancy of urinary tract or generalized malignancy of other system, and status post organ transplantation.

**Table 4:** Demographic characteristics and renal function of study and control group

	**Study group**	**Control group**
n	30	30
male/female (%)	12 (40%)/18 (60%)	12 (40%)/18 (60%)
mean age in years (range)	44.4 (20–72)	44.6 (20–76)
mean body mass in kg (range)	70.7 (50–100)	71.1 (50–91)
mean serum creatinine in μmol/l (range)	120.7 (38.1-388.9)	68.4 (45.8-114.4)
GFR (CKD-EPI formula) in ml/min (range)	66.8 (11–140)	102.2 (54–136)

The study protocol was approved by the local ethics committee. Informed consent was obtained from all participants. The study was performed in accordance with the Declaration of Helsinki Principles.

### Urine collection

Samples were collected from 30 patients and 30 healthy donors using a uniform protocol. The second or third-morning mid-stream urine was collected from all participants at a time of 1 and 3 hours after previous micturition. Sterile urine containers were used for the collection of samples. pH of the samples was stabilized at 7.2 by addition of 1/10^th^ vol. of 1 M HEPES pH 7.2 immediately after collection. Further sample preparation steps were carried out within 1 hour after collection during which the sample was kept at room temperature. Samples were vortexed for 2 minutes, centrifuged (3000xg, room temp.) for 10 minutes to clear the debris, filtered through the 0.4 μm filter (Rotilabo-Spritzenfilter, P819.1, Roth) and portioned into 1 ml aliquots, to avoid freeze/thaw cycles in repeated experiments of the same sample. Sample aliquots were stored at −80°C for further use. The protocol used follows the urine proteomic sample collection recommendations [17].

### Sample filtration

10 kDa cutoff membrane filters (Amicon Ultra-0.5, UFC501096, Millipore) were washed twice with MilliQ water prior to use. Urine was centrifuged through the membrane at 14000xg for 15 minutes. Next, 500 μl MQ was added to the retentate and centrifugation step was repeated. To recover the concentrated and desalted sample, the filter was placed upside down in a clean micro centrifuge tube and centrifuged for 2 minutes at 1000xg. The protein concentration was measured by the Bradford method. Aliquots of samples were stored at −80°C.

### Pooling samples and iTRAQ-labelled samples study design

When indicated, the aliquots (corresponding to 10 μg of protein) of 10 urine samples were pooled. Only samples from a single study group (disease or control) were pooled. 30 control (healthy) samples were divided into three control pooled samples (CPS’s I, II and III) and similarly, 30 ADPKD samples were divided into three disease pooled samples (DPS’s I, II and III). Age and sex matching was preserved within the three pairs of pooled sample groups. Three CPS’s and three DPS’s were obtained in two technical replicates (marked A and B) each, making a set of 12 pooled samples to be compared after iTRAQ labeling. As 4-plex iTRAQ was used, 2 CPS and 2 DPS samples were compared in one LC-MS/MS experiment. To analyze 12 samples we have carried out a set of 3 independent LC-MS/MS experiments. The study design is illustrated in Figure 1.

### iTRAQ labeling

Before labeling, protein aliquots were evaporated to dryness in a speedvac, dissolved in 20 μl Dissolution Buffer with 0.1% SDS, reduced with TCEP, cysteine-blocked with MMTS (reagents were provided with the iTRAQ kit from Applied Biosystems), and digested overnight with trypsin (Promega). The CPS and DPS samples were differentially labeled with one of the four iTRAQ tags (114, 115 for CPS samples and 116, 117 for DPS samples) for 1 h according to the iTRAQ manufacturer’s protocol. Next, the reaction was quenched by adding 100 μl H_2_O.

For each of the three LC-MS/MS experiments 2 CPS and 2 DPS iTRAQ-labeled samples were combined and 340 μl buffer was added [8 M urea, 0.2% IPG buffer pH 3–11 NL (GE Healthcare), 0.002% bromophenol blue in 50 mM Tris–HCl, pH 8.0]. The solution was applied to 18 cm IPG strip with 3–11 NL pH gradients (GE Healthcare) for isoelectrofocusing (IEF): 340 μl of sample/strip, corresponding to 400 μg protein. The IPG strip was rehydrated overnight in an IPG box (GE Healthcare). The next day, the strips were isoelectrofocused using a Ettan IPGphor 3 electrophoresis system (GE Healthcare) as follows. Two steps of electrophoresis were used. The first step consisted of a 5 h pre-run at 500 V. During this step, the conductivity decreases, and salts and other highly conductive compounds move towards the electrode (anode). Second, a long gradient focusing program was used: 1 h at 500 V, 9 h at 1000 V and 30 h at 8000 V (the final current was 5 μA).

After focusing, the strip was removed from the tray and the overlay oil was blotted with a paper tissue. Strip was wrapped in a parafilm and stored at −80°C. The strip was placed on a tray cooled with dry ice and cut into sections of ca. 7 mm. The sections were transferred into individual 1.5-ml siliconized Eppendorf tubes. In all, the 18-cm long gel strips were sliced into 26 sections. Peptides were extracted from gel sections by two cycles of adding 60 μl 0.1%TFA, 2% acetonitrile and vortexing the tubes for 40 minutes at room temperature. Aliquots with extracted peptides were stored at −80°C for LC-MS/MS analysis.

### Mass spectrometry - LC-MS/MS settings

The peptide mixture (20 μl) was applied to the nanoACQUITY UPLC Trapping Column (Waters) using water containing 0.1% formic acid as the mobile phase and then transferred to the nanoACQUITY UPLC BEH C18 Column (Waters, 75 μm inner diameter; 250-mm long) using an acetonitrile gradient (3–33% acetonitrile over 150 minutes) in the presence of 0.1% formic acid with a flow rate of 250 nl/min. The column outlet was directly coupled to the electrospray ion source of the LTQ-Orbitrap Velos mass spectrometer (Thermo Scientific) working in the regime of data-dependent MS to MS/MS switch. HCD fragmentation was used. Other Orbitrap parameters were as follows: one MS scan was followed by max. 5 MS/MS scans, capillary voltage was 1,5 kV, data were acquired in positive polarity mode.

### Mass spectrometry - Qualitative MS/MS data processing

The acquired MS/MS data were pre-processed with Mascot Distiller (version 2.3.2.0, Matrix Science, London, UK). The database search of the data using MASCOT search engine was carried out in a three-step procedure (described elsewhere [18], and in short in Additional file 2) to calculate MS and MS/MS measurements errors and to recalibrate the data for the repeated MASCOT search. The initial search parameters were set as follows: enzyme, semi-trypsin; fixed modification, cysteine modification by MMTS as well as iTRAQ labeling of the N-terminus of peptides and of lysine side chains; variable modifications - oxidation (M); max missed cleavages – 1, Swiss-Prot database with the taxonomy restricted to Homo sapiens (20273 sequences). For the repeated search the recalibrated data from all gel sections were merged into one input file and searched using MASCOT against a Swiss-Prot database supplemented with the decoy database to obtain the statistical assessment of the identification of each peptide by a joined target/decoy database search strategy [19]. This procedure provided *q*-value estimates for each peptide spectrum match (PSM) in the dataset. All PSMs with *q*-values > 0.01 were removed from further analysis. A protein was regarded as confidently identified if at least two peptides of this protein were found. Proteins identified by a subset of peptides from another protein were excluded from analysis. Proteins that exactly matched the same set of peptides were clustered into one group/cluster. MS/MS spectra of peptides meeting the above acceptance criteria were subjected to quantitative analysis step to obtain a list (Differential Protein List) of proteins differentially populated between a set of three CPS’s and three DPS’s.

### iTRAQ quantitative analysis

For protein quantitation only unique peptides (i.e. peptides belonging only to one protein/cluster) were included. In the first step, using MascotDistiller program iTRAQ reporter ion peaks were detected in the preprocessed MS/MS spectra; next, their intensities were corrected for isotope impurity using the information provided by the reagent manufacturer. For each spectrum a geometric mean of two reporter ion intensities belonging to one study group (CPS or DPS) were separately calculated. A ratio of these mean values (CPS mean divided by DPS mean) was reported as peptide ratio. If more than one spectrum was obtained for a peptide in a single LC-MS/MS experiment, median peptide ratio value from all spectra was used. Prior to the protein ratio calculations, peptide ratios were median-normalized to remove systematic bias. Proteins ratios were calculated as the median ratio of their peptide’s ratios. The statistical significance of a single protein ratio was assessed by an in house program Diffprot [20]. In this program the statistical validity of regulation/expression status of the protein represented by its calculated protein ratio is based solely on the statistical analysis of the set of all MS/MS datasets from a given experiment, without assumptions on the character of the distribution of peptide ratios in a dataset (e.g. its normality). In brief, the probability of obtaining a given protein ratio by a random selection from the dataset is tested by multiple rounds of protein ratio calculation for a large number of permuted decoy datasets in which the peptide-protein assignment has been scrambled. Calculated *p*-values were adjusted for multiple testing using a FDR-controlling procedure, yielding protein ratio q-values reported in Table 2.

### Quantitative analysis of selected proteins using multiple reaction monitoring

We have selected a subset of proteins from the Differential Protein List shown in Table 2 for further analysis of non-pooled, individual samples using the multiple reaction monitoring (MRM) technique, used in conjunction with stable-isotope-labeled peptide standards (SIS). The presence of natural MRM transitions for peptides from 17 proteins was first checked in samples of urine collected separately from healthy volunteers. Only for nine proteins the natural transitions corresponding to selected peptides yielded satisfactory results and SIS peptides were generated for these. The transitions for peptides corresponding to the remaining eight proteins could not be detected with a sufficient signal to noise ratio.

Finally a set of peptides, corresponding to nine proteins, used for further MRM analysis of individual ADPKD and control samples, consisted of 14 peptides:

1. Antithrombin-III – peptide: TSDQIHFFFAK, peptide: FATTFYQHLADSK

2. Cystatin-M - peptide: DLSPDDPQVQK, peptide: AQSQLVAGIK

3. Transthyretin – peptide: AADDTWEPFASGK

4. Retinol-binding protein 4 – peptide: YWGVASFLQK, peptide: DPNGLPPEAQK

5. Proactivator polypeptide – peptide: EIVDSYLPVILDIIK, peptide: LVGYLDR

6. Apolipoprotein A-IV – peptide: SELTQQLNALFQDK, peptide: LLPHANEVSQK

7. Complement C3 – peptide:TIYTPGSTVLYR

8. Histidine-rich glycoprotein – peptide: DGYLFQLLR

9. Myocilin – peptide: YELNTETVK

Specific tryptic peptide sequences, to be used for SIS peptide synthesis, were selected for the nine proteins based on the number of observations in the PeptideAtlas MS/MS database ( http://www.peptideatlas.org/, Institute for Systems Biology, Seattle, WA), and on specific criteria required in SIS peptides such as length and lack of amino acid modifications [21,22]. SIS peptides were synthesized by JPT Peptide Technologies GmbH using the SpikeTides_L option (JPT Peptide Technologies GmbH, Berlin, Germany) using isotopically labeled C-terminal amino acids, ^13^C_6_^15^ N_2_-Lys (98% isotopic enrichment) or ^13^C_6_^15^ N_4_-Arg (98% isotopic enrichment). MRM analysis was performed using a Waters Xevo TQ mass spectrometer (Waters, MA, USA) coupled to a Waters nanoAcquity UPLC via a Zspray Nanoflow source with a 10 μm SilicaTip PicoTip emitter (New Objective, MA, USA). Mobile phase A was 0.1% formic acid (Sigma-Aldrich) in LC-MS grade water (J.T.Baker, Netherlands), and mobile phase B was LC-MS grade acetonitrile (J.T.Baker) with 0.1% formic acid. Peptides were loaded onto a Waters Symmetry C18 pre-column (180 μm x 20 mm, 5 μm particle size) and separated using a 40 min LC run, with a 27 min gradient of mobile phase B changing from 1 to 45% on a Waters nanoAcquity UPLC BEH130 C18 Column (100 μm x 100 mm, 1.7 μm particle size). Other MS instrument parameters included a capillary voltage of 3.1 kV, a purge gas flow of 100 L/h, cone gas flow of 5 L/h, NanoFlow gas set at 1.0 Bar, and a source temperature of 150°C. MRM parameters were empirically optimized using pure SIS peptides to generate the highest possible signal for each individual peptide and resulting ion fragments. The optimal charge state and optimal cone voltage were determined for each SIS peptide by injecting 1 pmol (in 0.1% formic acid) on-column and ramping the cone voltage from 20 to 70 V in 5 V steps while gating all the possible parent ion charge states (2+, 3+, 4+) using the selected ion recording (SIR) function controlled by the Waters MassLynx V4.1 software. The daughter ions generating the highest possible signal and their individual, optimal collision energy (CE) voltages were determined empirically by injecting 1 pmol (in 0.1% formic acid) of SIS peptide on-column and ramping the CE voltage up and down five 2 V steps from that suggested by the Skyline Ver. 1.3 software (University of Washington, MacCoss Lab, Department of Genome Sciences, UW) for the Waters Xevo instrument. All possible b- and y-series fragment ions for both 2+ and 3+ precursor ion charge states spanning a *m/z* range from 300 to 1500 were tested. MRM scans for optimization of MRM Q1/Q3 ion pairs were conducted with the optimized cone voltages with the Span setting set to 0 and with dwell times of 10 milliseconds for each transition. From this data, using the Skyline Ver. 1.3 software, the 5 transitions that produced the strongest signals were selected on a per-peptide basis, with a preference toward higher-mass y series ions if the abundances were similar. These top 5 transitions were then checked for signal interferences when present in a sample-digest background. The SIS peptide mix was analyzed by LC-MRM/MS using transitions for heavy (SIS) and natural (endogenous) peptides, both in buffer and in a sample digest. Identical MRM acquisition parameters were used for the heavy and natural forms of each peptide, while taking into account the Q1/Q3 mass differences due to the stable-isotope label. The transitions that maintained the same relative intensities in both the buffer and sample were considered as interference free. This analysis is also used to determine the retention time as well as confirm the identity of the ion signals observed for natural and heavy peptides, thus verifying the identity of the natural peptides which co-elute with the corresponding SIS peptides.

MRM analysis was performed on a new set of 52 samples (27 ADPKD *vs.* 25 healthy controls), with an injection volume of 4 μl resulting in 2 μg of protein digest on-column. Samples were prepared basically as for iTRAQ pooling analysis, see Sample filtration section. First, from each sample, an aliquot of protein fraction containing 10 μg of total protein was transferred to silanized vials. Then the volume of each sample was brought to 30 μl using 100 mM solution of NH_4_HCO_3_. 100 mM DTT (Sigma Cat no D8161-5 G) was added to the samples to the final concentration of 10 mM and incubated at 56°C for 40 min. To block reduced cysteines 0.5 M iodoacetamide (Sigma Cat no I1149-5 G) to the final concentration of 50 mM was used and the sample was incubated at room temperature for 30 minutes in darkness. Trypsin (Promega cat. no V511A) was added to samples in 1:20 vol./vol. ratio and incubated at 37°C overnight. Finally, trifluoroacetic acid was added to digested protein samples to reduce pH to 2 and inactivate trypsin.

Peptide standards were added to the samples post digestion as a SIS mixture in which individual SIS peptides were balanced to obtain at least a ratio of 1:10 between the endogenous natural peptide and the corresponding SIS peptide in a positive sample. All MRM data was processed using the Skyline Ver. 1.3 software with default values for peak integration and Savitzky-Golay peak smoothing. All integrated peaks were manually inspected to ensure correct peak detection and accurate integration. All peptides were targeted using 5 MRM ion pairs per peptide unless an interference was found in a transition then reducing that number to four transitions per peptide. The integrated peak areas for the individual transitions detecting the 4–5 ion fragments per peptide were summed. The relative protein amounts in the samples are reported as Peak Area Ratios To Heavy, which refers to the ratio of the integrated area of the endogenous (natural) peak to the integrated area of the corresponding standard (SIS) peptide.

## Abbreviations

ADPKD: Autosomal Dominant Polycystic Kidney Disease; ESRD: End Stage Renal Disease; DPS: Disease Pooled Samples; CPS: Control Pooled Samples; DPL: Differential Protein List; SIS peptides: stable-isotope-labeled internal standard peptides.

## Misc

Magda Bakun and Mariusz Niemczyk contributed equally to this work

## Competing interests

The authors declare that they have no competing interest.

## Authors’ contribution

M.B. – optimized peptide IEF, carried out LC-MS experiments, analyzed LC-MS data. M.N. – did patients selection, characterization, enrollment to the study, sample collection, wrote the manuscript. S.N. - did patients selection, characterization, enrollment to the study. A.F. – optimized sample prep protocol, carried out LC-MS experiments, M.K. – provided statistical analysis of the data. D.D., R.J. – designed MRM experiments, selected peptides, optimized MRM conditions. A.P., A.B. – carried out MRM experiments, analyzed data. M.D. - designed the study, wrote manuscript. L.P. - designed the study, established exclusion/inclusion criteria, wrote manuscript. All authors read and approved the final manuscript.

## Supplementary Material

Additional file 1 List of 1 700 proteins identified by at least two peptides. Click here for file

Additional file 2 Mass measurement error correction and identification estimation q-value. Click here for file

## References

[B1] Chang MY Ong ACM Autosomal dominant polycystic kidney disease: recent advances in pathogenesis and treatment Nephron Physiol 2008 108 p1 p7 10.1159/000112495 18075279

[B2] Torres VE Meijer E Bae KT Rationale and design of the TEMPO (Tolvaptan Efficacy and Safety in Management of Autosomal Dominant Polycystic Kidney Disease and its Outcomes) 3–4 Study Am J Kidney Dis 2011 57 5 692 699 10.1053/j.ajkd.2010.11.029 21333426

[B3] Walz G Budde K Mannaa M Everolimus in patients with autosomal dominant polycystic kidney disease N Engl J Med 2010 363 9 830 840 10.1056/NEJMoa1003491 20581392

[B4] Serra AL Poster D Kistler AD Sirolimus and kidney growth in autosomal dominant polycystic kidney disease N Engl J Med 2010 363 9 820 829 10.1056/NEJMoa0907419 20581391

[B5] Hogan MC Masyuk TV Page LJ Randomized clinical trial of long-acting somatostatin for autosomal dominant polycystic kidney and liver disease J Am Soc Nephrol 2010 21 6 1052 1061 10.1681/ASN.2009121291 20431041 PMC2900957

[B6] Julian BA Wittke S Haubitz M Urinary biomarkers of IgA nephropathy and other IgA-associated renal diseases World J Urol 2007 25 467 10.1007/s00345-007-0192-5 17619884

[B7] Mason SB Lai X Bacallao RL The biomarker enriched proteome of autosomal dominant polycystic kidney disease cyst fluid Proteomics Clin Appl 2009 3 1247 1250 10.1002/prca.200800163 20526430 PMC2880522

[B8] Kistler AD Mischak H Poster D Identification of a unique urinary biomarker profile in patients with autosomal dominant polycystic kidney disease Kidney Int 2009 76 89 96 10.1038/ki.2009.93 19340089

[B9] Nagaraj N Mann M Quantitative analysis of the intra- and inter- individual variability of the normal urinary proteome J Proteome Res 2011 10 2 637 645 10.1021/pr100835s 21126025

[B10] Marimuthu A O'Meally RN Chaerkady R A comprehensive map of the human urinary proteome J Proteome Res 2011 10 6 2734 2743 10.1021/pr2003038 21500864 PMC4213861

[B11] Adachi J Kumar C Zhang Y The human urinary proteome contains more than 1500 proteins, including a large proportion of membrane proteins Genome Biol 2006 7 R80 10.1186/gb-2006-7-9-r80 16948836 PMC1794545

[B12] Kentsis A Monigatti F Dorff K Urine proteomics for profiling of human disease using high accuracy mass spectrometry Proteomics Clin Appl 2009 3 1052 1061 10.1002/prca.200900008 21127740 PMC2994589

[B13] Li QR Fan KX Li RX A comprehensive and non-prefractionation on the protein level approach for the human urinary proteome: touching phosphorylation in urine Rapid Commun Mass Spectrom 2010 24 823 832 10.1002/rcm.4441 20187088

[B14] Allen E Piontek KB Garrett-Mayer E Loss of polycystin-1 or polycystin-2 results in dysregulated apolipoprotein expression in murine tissues via alterations in nuclear hormone receptors Hum Mol Genet 2006 15 11 21 16301212 10.1093/hmg/ddi421 PMC1525254

[B15] Zhou J Ouyang X Ciu X Renal CD14 expression correlates with the progression of cystic kidney disease Kidney Int 2010 78 550 560 10.1038/ki.2010.175 20555320 PMC3422025

[B16] Pei Y Obaji J Dupuis A Unified criteria for ultrasonographic diagnosis of ADPKD J Am Soc Nephrol 2009 20 1 205 212 10.1681/ASN.2008050507 18945943 PMC2615723

[B17] Thongboonkerd V Practical points in urinary proteomics J Proteome Res 2007 6 3881 3890 10.1021/pr070328s 17824635

[B18] Mikula M Gaj P Dzwonek K Comprehensive analysis of the palindromic motif TCTCGCGAGA: a regulatory element of the HNRNPK promoter DNA Res 2010 17 245 260 10.1093/dnares/dsq016 20587588 PMC2920758

[B19] Elias JE Gygi SP Target-decoy search strategy for increased confidence in large-scale protein identifications by mass spectrometry Nat Methods 2007 4 207 214 10.1038/nmeth1019 17327847

[B20] Malinowska A Kistowski M M. Diffprot - software for non-parametric statistical analysis of differential proteomics data J Proteomics 2012 75 4062 4073 10.1016/j.jprot.2012.05.030 22641154

[B21] Domanski D Smith DS High-flow multiplexed MRM-based analysis of proteins in human plasma without depletion or enrichment Clin Lab Med 2011 31 3 371 384 10.1016/j.cll.2011.07.005 21907103

[B22] Parker CE Domanski D Mass Spectrometry in High-Throughput Clinical Biomarker Assays: Multiple Reaction Monitoring Top Curr Chem 2012 2012 10.1007/128_2012_353 22886709

