# Supplementary material for: Urine proteome of autosomal dominant polycystic kidney disease patients
Source: Clin Proteomics. 2012 Dec 11;9(1):13. doi: 10.1186/1559-0275-9-13 (PMC3607978; doi:10.1186/1559-0275-9-13)
Supplement: Additional file 2 — Mass measurement error correction and identification estimation q-value. [file 1559-0275-9-13-S2.doc]

**Mass measurement error correction**

The maximum mass deviation (MMD) for precursor and fragment ions were established in a procedure involving two database searches separated by a mass recalibration step. Data from each LC-MS/MS run was searched in a first pass with permissive MMD settings (precursor ions: ±40 ppm, fragment ions: ±0.8 Da). The resulting Mascot DAT files were next imported to an in-house Java tool (DATViewer, http://proteom.ibb.waw.pl/datviewer/index.html) which implements a procedure for systematic mass errors elimination and high accuracy MMD estimation. Only PSMs with a score value exceeding the smaller of the Mascot identity and homology thresholds were used for the mass accuracy assessment. For each LC-MS/MS run the mass correction was performed by fitting a smooth LOESS curve to the scatterplot of the observed mass errors versus precursor ions masses. The obtained normalization function was then used for the entire set of precursor ions. A separate calibration function was also calculated for fragmentation spectra on the basis of the masses of singly charged y-series ions. The new MMD values were estimated as three-fold the standard deviation of the mass errors after recalibration. Finally, the mass-corrected spectra were exported as Mascot Generic File format files and resubmitted to the database search engine in order to obtain final peptide and protein identifications.

**Identification estimation q-value**

For statistical assessment of peptide assignments the fragmentation spectra were matched against a joined target/decoy database [1] The target part of the database containing true protein sequences was concatenated with a decoy part composed of reversed versions of these sequences. All peptide PSMs, from the forward and the reverse database, were sorted according to their score modified by subtracting the smaller of the Mascot identity and homology thresholds. The number of false positive identifications associated with a specific position P in the sorted list was estimated by doubling the number PSMs from the decoy part of the database at positions not greater than P. The position-related false discovery rate (FDR) was calculated by dividing the estimated number of false positives by the total number of PSMs at preceding positions. To address the fact that FDR itself is not a function of the underlying score (i.e. FDR can decrease with increasing position in the sorted list), the position-related FDRs were next converted to q-values, as described in [2]. The presented analysis was performed using a proprietary software tool implemented in Java programming language (MScan, http://proteom.ibb.waw.pl/mscan/index.html).

1. Elias JE, Gygi SP. Target-decoy search strategy for increased confidence in large-scale protein identifications by mass spectrometry. *Nature methods* 2007; 4: 207-214.

2. Kall L, Storey JD, MacCoss MJ, Noble WS. Assigning significance to peptides identified by tandem mass spectrometry using decoy databases. *Journal of proteome research* 2008; 7: 29‑34.
